# Clinical-Pathological Conference Series from the Medical University of Graz: Case No 161: A 42-year-old journalist with fatigue, elevated liver function tests, hyperglycemia and pruritus

**DOI:** 10.1007/s00508-018-1379-z

**Published:** 2018-08-21

**Authors:** Elisabeth Fabian, Markus Peck-Radosavljevic, Elisabeth Krones, Helmut Mueller, Caroline Lackner, Christopher Spreizer, Csilla Putz-Bankuti, Werner Fuerst, Nora Wutte, Peter Fickert, Hansjörg Mischinger, Guenter J. Krejs

**Affiliations:** 1 Division of Gastroenterology and Hepatology, Department of Internal Medicine III, Medical University of Vienna, Vienna, Austria; 2 Department of Internal Medicine and Gastroenterology, Klinikum Klagenfurt am Wörthersee, Klagenfurt, Austria; 3 Division of Gastroenterology and Hepatology, Department of Internal Medicine, Medical University of Graz, Auenbruggerplatz 15, 8036 Graz, Austria; 4 Division of Transplant Surgery, Department of Surgery, Medical University of Graz, Graz, Austria; 5 Department of Pathology, Medical University of Graz, Graz, Austria; 6 Division of General Radiology, Department of Radiology, Medical University of Graz, Graz, Austria; 7 Department of Internal Medicine, Hörgas-Enzenbach Hospital, Gratwein-Straßengel, Austria; 8 Department of Internal Medicine, Leoben Hosptial, Leoben, Austria; 9 Department of Dermatology and Venerology, Medical University of Graz, Graz, Austria; 10 Division of General Surgery, Department of Surgery, Medical University of Graz, Graz, Austria

**Keywords:** Autoimmune pancreatitis type 1, IgG4, Cholestasis, Total pancreatectomy, Leukocytoclastic vasculitis

## Presentation of case

### Dr. C. Putz-Bankuti:

The patient, a 42-year-old white male, had been admitted to another teaching hospital for renal colic and subsequent shockwave lithotripsy 2 years before first presenting at the Outpatient Clinic for Gastroenterology and Hepatology at the University Medical Center in Graz. At that time, elevated liver function tests had been known for years and were attributed to hepatic steatosis: total bilirubin 2.5 mg/dl (normal: 0.1–1.2 mg/dl), direct bilirubin 1.8 mg/dl (normal: 0–1.0 mg/dl), alkaline phosphatase 813 U/l (normal: 35–105 U/l), gamma-glutamyl transferase (GGT) 1153 U/l (normal: <38 U/l), aspartate amino transferase (AST) 581 U/l (normal: <30 U/l), alanine amino transferase (ALT) 1294 U/l (normal: <35 U/l), lactate dehydrogenase (LDH) 334 U/l (normal: 120–250 U/l). Computed tomography (CT) of the abdomen revealed liver parenchymal fibrosis and steatosis, cholecystolithiasis, localized thickening of the wall of the common bile duct probably following previous cholangitis and enlarged lymph nodes along the celiac trunk and the transverse fissure.

The patient was again admitted to the same hospital for fatigue one year before the current admission for elevated liver function tests and hyperglycemia. Physical examination was unremarkable (weight 71 kg, height 170 cm, pulse 80/min, blood pressure 100/70 mm Hg), but the patient complained of pruritus. Laboratory data: erythrocytes 4.4 T/l (normal: 4.50–5.90 T/l), hemoglobin (Hb) 13.2 g/dl (normal: 12.0–15.3 g/dl), hematocrit 36.5% (normal: 35.0–45.0%), eosinophils 9.9% (normal: <5%), AST 659 U/l, ALT 981 U/l, GGT 2030 U/l, AP 1260 U/l, LDH 385 U/l, total bilirubin 2.75 mg/dl, glucose 529 mg/dl (normal: 70–115 mg/dl), HbA1c 10% (normal: 4.3–5.9%), ferritin 530 ng/ml (normal: 30–400 ng/ml). Further laboratory data were all normal or negative: creatinine kinase, serum myoglobin, C‑reactive protein, total protein, albumin, iron, transferrin, transferrin saturation, prothrombin time and partial thromboplastin time, thyroid stimulating hormone, prostate specific antigen, alpha1-antitrypsin, ceruloplasmin, immunoglobulin (Ig) M, IgG4, antinuclear antibodies (ANA), smooth muscle antibodies (SMA), liver-kidney microsomal antibodies (LKM) and anti-mitochondrial antibodies (AMA). Hepatitis A and B antibodies were positive due to vaccination but other hepatitis markers were negative. Endocrinological parameters: proinsulin was increased (41.9 pmol/l, normal: <8 pmol/l), insulin, C‑peptide, antibodies against insulin, tyrosine phosphatase and glutamic acid decarboxylase 65 were normal.

The medical history further revealed meningitis in his early childhood with hydrocephalus, shunt implantation with later revision, surgery for strabismus, tonsillectomy, and surgery following right shoulder trauma. The family history was positive for diabetes mellitus type II in the patient’s father.

Magnetic resonance cholangiopancreatography (MRCP) and magnetic resonance imaging (MRI) showed moderate distension of the intrahepatic bile ducts in the left lobe of the liver with irregular contours, localized beading, and alternating strictures and dilatations. Distally increasing stenosis and some areas of complete luminal obliteration were noted. Multiple stenoses of the common bile duct with only faint visualization were present in its proximal portion. The gallbladder appeared unremarkable, the liver parenchyma was homogeneous without space-occupying lesions, there was mild splenomegaly (length 14 cm) and moderate lymphadenopathy along the hepatoduodenal ligament (maximum diameter 23 mm). Endoscopic retrograde cholangiopancreatography (ERCP) showed an edematous ampulla of Vater and an irregular pancreatic duct repeatedly releasing white viscous secretion. A proton pump inhibitor was prescribed for multiple fibrin-covered ulcers in the distal esophagus. Liver biopsy was compatible with primary sclerosing cholangitis (PSC) and there was no evidence of an overlap syndrome. Subsequently, the patient was treated with ursodeoxycholic acid, cholestyramine, an antihistamine, a selective serotonin reuptake inhibitor (SSRI) and insulin (HbA1c decreased to 5.8%). Under this therapy liver function improved, but values were still significantly elevated: bilirubin 1.0 mg/dl, AP 555 U/l, GGT 392 U/l, AST 128 U/l, ALT 262 U/l.

**Fig. 1:**
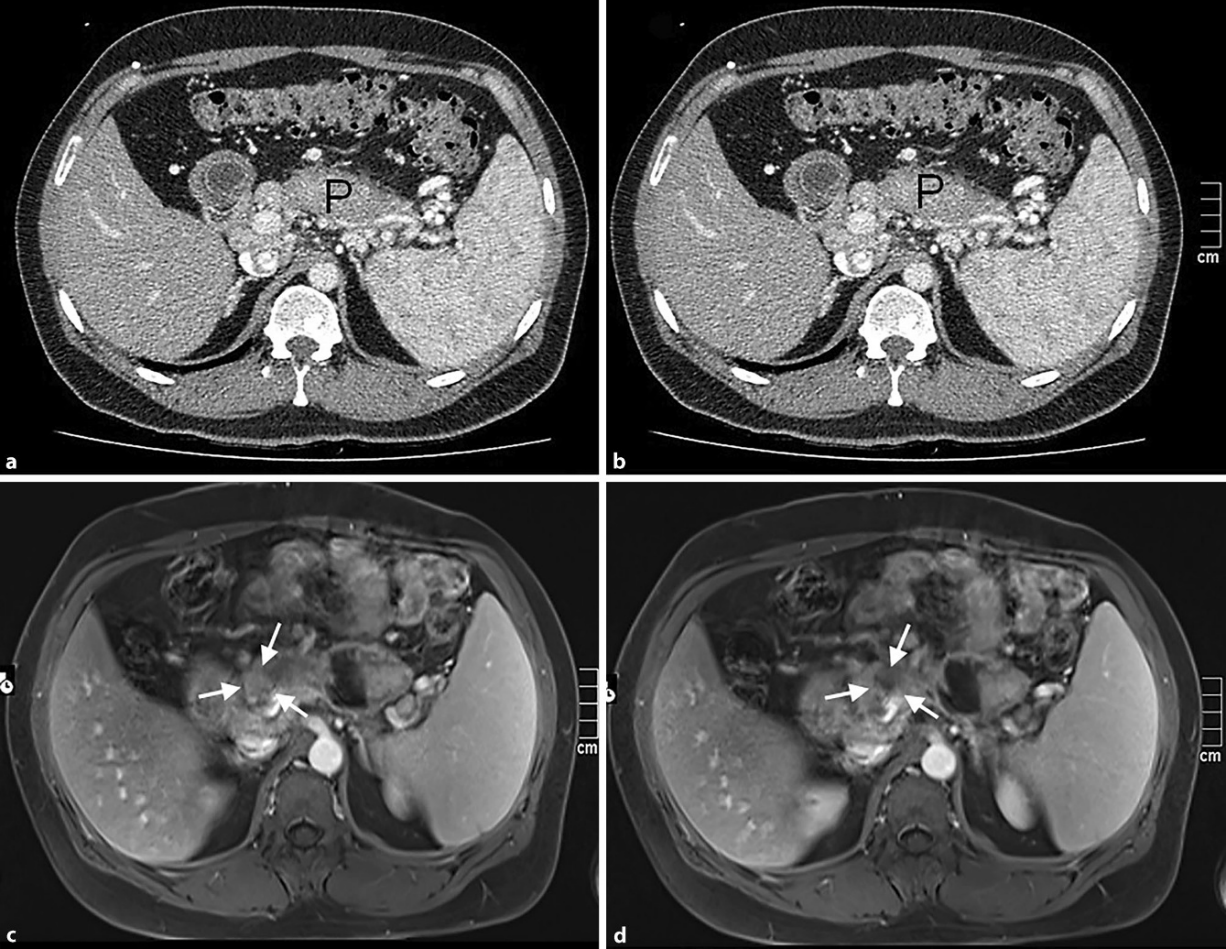
**a,b** Contrast-enhanced CT showing diffuse enlargement of the body of the pancreas (*P*) with loss of lobular structure. **c,d** T1-weighted fat-saturated contrast-enhanced magnetic resonance images showing an ill-defined hyopodense lesion (*arrows*) in the head of the pancreas. These findings were interpreted as being consistent with a carcinoma (or cystadenocarcinoma) of the head of the pancreas with diffuse infiltration of the body of the pancreas

The patient sought a second opinion when someone brought up the subject of liver transplantation. Since the consulted physician did not see an indication for liver transplantation and the patient had not responded to ursodeoxycholic acid therapy, he was referred to the University Medical Center in Graz to be enrolled in a clinical trial studying 24-nor-ursodeoxycholic acid in patients with PSC. Except for several bland diverticula, colonoscopy was unremarkable. Follow-up MRCP and MRI showed slight accentuation of the first-order intrahepatic bile ducts but no significant biliary retention. The common bile duct was thin with slight periductal fibrosis but was not beaded. The pancreatic duct appeared unremarkable. Contrast enhancement showed a subsepted pseudocyst (7.0 × 3.6 cm) primarily localized in the tail of the pancreas but also extending to the omental bursa. The pancreas appeared grossly altered (Fig. 1) and several enlarged lymph nodes (diameter up to 1 cm) were visible along the mesentery. A panel of genetic tests was negative for mutations causing hereditary pancreatitis. Tumor markers including CA 19-9, CEA and CA 72-4 were unremarkable.

The further management of the patient led to the diagnosis.

## Differential diagnosis

### Dr. M. Peck-Radosavljevic:

This complex case of a probably liver-associated disease was first recognized when the patient was admitted to hospital due to renal colic. At that time, elevated liver function tests had been known for years. Thus, it is not surprising that abdominal CT revealed pathological changes of the liver, such as damage to the liver parenchyma consistent with steatosis and fibrosis, cholecystolithiasis and local thickening of the wall of the common duct. After this first hospitalization the patient’s medical history was unremarkable for 1 year, but he then had to be readmitted for fatigue, elevated liver function tests and hyperglycemia. Cholestasis had increased and hyperglycemia was prominent. Increased serum ferritin could be due to hemochromatosis causing both hepatic abnormalities and diabetes; however, hemochromatosis can be ruled out here because other iron parameters, such as transferrin and transferrin saturation, which are typically increased in iron storage diseases, were normal. Further laboratory data focusing on liver disease (e.g. albumin, C‑reactive protein, prothrombin time, thyroid stimulating hormone, alpha1-antitrypsin and ceruloplamsin) were also normal. Thus, it can be suggested that in the presence of cholestasis, the metabolic function of the liver had hardly been affected by the underlying disease. The IgM, IgG4, ANA and SMA levels were normal, so there was no indication of autoimmune liver disease.

According to the protocol, MRCP showed moderate distension of the intrahepatic bile ducts in the left lobe of the liver with irregular contours, localized beading, and alternating strictures and dilatations. The narrowing of the common bile duct that was more prominent in the central portion could explain the failure to visualize it by ERCP. The ERCP further detected an edematous papilla and an irregular pancreatic duct repeatedly releasing white viscous secretion, which might be an important clue for the underlying problem in this case. Histopathology of the liver biopsy revealed PSC and ruled out an overlap syndrome; however, in PSC one would expect higher inflammatory markers than found in this patient. Actually, laboratory data are more compatible with an overlap syndrome than with PSC in this case, but this was not confirmed by liver histology. The patient received ursodeoxycholic acid for PSC, cholestyramine, an antihistamine and an SSRI for treatment of pruritus, along with insulin for hyperglycemia. Under this treatment blood glucose, cholestasis, inflammation and liver function tests significantly improved, and according to the MRCP/MRI report, the beaded configuration of bile ducts disappeared; however, after this treatment the pancreas appeared grossly abnormal and contained a subsepted cyst, presumably a pseudocyst.

In summary, this case is very complex and there is no specific finding suggesting a definite diagnosis. Differential diagnosis of elevated liver function tests includes a wide range of diseases (Table 1); however, chronic hepatitis C and B, acute viral hepatitis, steatosis/steatohepatitis, hemochromatosis, alpha1-antitrypsin deficiency and Wilson’s disease can be ruled out as diagnoses because they were not confirmed by laboratory data. According to the protocol, hepatotoxic substances (medication/toxins) can also be excluded. Autoimmune hepatitis may sometimes cause significantly elevated liver function tests and cholestasis (overlap syndrome), as present here. Negative serology does not necessarily rule out the disease but also does not really favor a suspicion of autoimmune hepatitis. The ALT level can also be elevated due to celiac disease, but this can be excluded because of the negative medical history for this disease. Since this situation was not AST-predominant, alcohol-related liver injury, steatosis/steatohepatitis and cirrhosis can be also ruled out as diagnoses. Non-hepatic causes given in Table 1 are also unlikely to have caused the patient’s problems, and the medical history does not hint at an ischemic event. Although the bile duct had been obstructed, an acute bile duct obstruction could not have been present because cholestasis was not as severe as expected in acute biliary obstruction and bilirubin was not as high as it usually is in this situation. Finally, nothing in the protocol suggests acute Budd-Chiari syndrome. Taking into account additional diagnoses that are related to elevated alkaline phosphatase (Table 1), primary biliary cirrhosis, infiltrating diseases of the liver, hepatic metastases, vanishing bile duct syndrome and benign recurrent cholestasis can be excluded due to lack of evidence. Although the patient was diagnosed with PSC, the rapid and significant improvement of cholestasis and liver function tests under treatment is very unusual for PSC and makes the diagnosis highly questionable. Except for infection, there is no evidence that the non-hepatic causes of elevated alkaline phosphatase summarized in Table 1 were present in this case. Laboratory data indicated increased eosinophils, possibly due to a parasitic infection. This suggestion is further strengthened by the diagnosis of splenomegaly, which among other causes such as portal hypertension (not present here) can be due to infections. Moderately enlarged lymph nodes along the portal area may also suggest more an infectious than a malignant cause of lymphadenopathy. The finding of a subsepted cyst in the pancreas provides an additional clue for the diagnosis of parasitic infection leading to cyst formation, i.e. echinococcosis, also known as hydatid disease or hydatidosis. Echinococcosis is caused by larval infection with *Echinococcus granulosus *and is characterized by long-term growth of metacestode (hydatid) cysts, occurring mainly in the liver and lungs but also in other organs of the intermediate host [1]. Hydatid cysts can be classified differently according to their appearance [2]. Subsepted cysts may be due to formation of daughter cysts that are frequent in echinococcosis [3].

**Table 1:** Differential diagnoses of elevated liver function tests [4]

Moderate elevation of transaminases (<5 × ULN)	Significant elevation of transaminases (>15 × ULN)	Elevation of alkaline phosphatase
*Hepatic: ALT-predominant*	Acute viral hepatitis (A–E, herpes)	*Hepatobiliary*
Chronic hepatitis C	Medication/toxins	Bile duct obstruction
Chronic hepatitis B	Ischemic hepatitis	Primary biliary cirrhosis
Acute viral hepatitis (A-E, EBV, CMV)	Wilson’s disease	Primary sclerosing cholangitis
Steatosis/steatohepatitis	Acute bile duct obstruction	Medications
Hemochromatosis	Acute Budd-Chiari syndrome	Infiltrating diseases of the liver
Medication/toxins	Hepatic artery ligation	Hepatic metastasis
Autoimmune hepatitis		Hepatitis
Alpha1-antitrypsin deficiency		Cirrhosis
Wilson’s disease		Vanishing bile duct syndromes
Celiac disease		Benign recurrent cholestasis
*Hepatic: AST-predominant*		*Non-hepatic*
Alcohol-related liver injury		Bone disease
Steatosis/steatohepatitis		Pregnancy
Cirrhosis		Chronic renal failure
*Non-hepatic*		Lymphoma/other malignancies
Hemolysis		Congestive heart failure
Myopathy		Childhood growth
Thyroid disease		Infection/inflammation
Strenuous exercise		
Macro-AST		

*ULN* upper limit of normal, *EBV* Epstein-Barr virus, *CMV* cytomegaly virus, *AST* aspartate-amino transferase, *ALT* alanine-amino transferase

## Dr. M. Peck-Radosavljevic’s diagnosis

Echinococcosis with hydatid cyst (with daughter cysts) in the pancreas.

## Discussion of case

### Dr. P. Fickert:

When the patient was referred to the outpatient clinic of the Division of Gastroenterology and Hepatology at the University Medical Center in Graz, there was no evidence for pancreatitis in his medical history, while the radiologic findings revealed a subsepted pancreatic cyst of unclear origin. Thus, different causes such as intraductal papillary mucinous neoplasia (IPMN), probably associated with autoimmune pancreatitis (AIP), and mucous cystadenoma or carcinoma were considered. The finding of repeated release of white viscous secretion from the pancreatic duct actually did make us think of a mucous cystadenoma or carcinoma; however, the intermittent cholestasis resulting in significantly elevated liver function tests is atypical for cystadenoma or carcinoma and could also be due to different hepatic diseases as already discussed by Dr. Peck-Radosavljevic. Cyst formation in AIP is rare. Currently, only a small number of cases have been reported [5–10]. In the patient under discussion no specific diagnostic criteria for AIP were found; however, this does not necessarily rule out the diagnosis. An IPMN may be rarely associated with AIP [11–15]. Thus, it was thought that only histology would lead to the final diagnosis in this case. Performing transabdominal or transgastric biopsy of the cystic lesion was, however, not advisable because of vascular convolutes in the vicinity. Furthermore, adhesions of intestinal loops as documented in this patient are typical for tumors.

Since the radiologic findings did not provide a clear diagnosis and biopsy was not possible, the attending physicians suggested laparotomy to obtain tissue from the cystic lesion and to establish the diagnosis histologically.

### Dr. W. Fuerst:

Dr. Peck-Radosavljevic suspected echinococcosis; however, parasite screening was negative.

### Dr. G.J. Krejs:

Parasite screening tests are known to be negative sometimes, even though the patient is infected. Recently, we saw a case where the results had been negative for hookworm (*Ancylostoma duodenale*) three times although the patient was infected. Screening should always be repeated when a parasitic infection is suspected, even when one or two tests have been negative.

### Dr. H. Mueller:

**Fig. 2:**
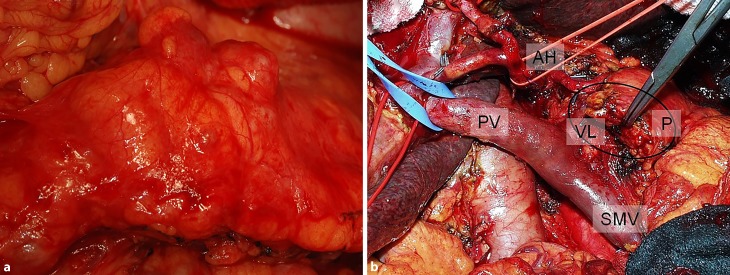
**a** Intraoperative view of the body of the pancreas. An inflammatory fibrotic capsule with adjacent fatty tissue covers the pancreas, which appears to harbor multiple small masses and cysts. **b** Intraoperative situs after resection of the proximal pancreas with (*P*) residual pancreatic tail with clamped main pancreatic duct, superior mesenteric vein (*SMV*), portal vein (*PV*), splenic vein (*VL*) and common hepatic artery (*AH*)

Prior to surgery, the finding of a subsepted pancreatic pseudocyst of unclear origin by MRI was additionally investigated by CT, which suggested a cystic primary neoplasm, either cystadenoma or cystadenocarcinoma of the pancreas. At surgery, the chronically inflamed pancreas was found grossly enlarged with loss of its lobulated contour (“sausage-shaped”), very hard and in some areas shaped irregularly because of connective tissue attached to the pancreatic surface (Fig. 2). Macroscopically, the pancreas suggested carcinoma to the surgeon; however, intraoperative frozen sections revealed non-neoplastic chronic pancreatitis. Due to the discrepancy between macroscopic and microscopic appearance and the fact that the patient had already developed insulin-dependent diabetes, a total pancreatectomy was performed.

### Dr. P. Fickert:

In this case both MRI and CT could not clearly distinguish chronic pancreatitis from an intraductal pancreatic neoplasm, cystadenoma or carcinoma, which may sometimes even occur concomitantly. Recently, Roch et al. found that IPMNs are associated with a significantly higher rate of autoimmune diseases than expected in the general population [16]. About 82% of patients with AIP also have pancreatic intraepithelial neoplasms (Pan-IN), suggesting that AIP might be associated with an elevated risk for malignancy [17]. Furthermore, about 10% of cases with pancreatic ductal adenocarcinoma additionally show histological features of AIP [18]. Shiokawa et al. found that in some patients AIP develops as a paraneoplastic syndrome and described dermatomyositis as a concomitant condition [19]. Among the few reported cases of concurrent AIP and pancreatic cancer, some described AIP surrounding the cancer, whereas other reports described AIP in sites remote from the cancer. Further investigation is needed to determine whether AIP induces pancreatic cancer due to chronic inflammation, Pan-IN leads to AIP-like stromal tissue, or the occurrence of concurrent Pan-IN and AIP is coincidental [20]. Histological examination is essential to clearly distinguish AIP from intraductal pancreatic neoplasia, cystadenoma or carcinoma and possible overlaps. Surgery had to be performed in this case because biopsy of the suspected mass could not be done transabdominally or transgastrically for the reasons mentioned above. Resected specimens were examined histologically by Dr. Lackner.

### Dr. C. Lackner:

Macropscopically, the resected specimens of the duodenum and the grossly fibrotic pancreas were 29 × 3.8 cm and 12 × 5.5 × 3.6 cm in size. The pancreatic head was 6 × 4 × 3.6 cm and showed a whitish grey mass (2.5 × 2.5 × 2 cm) with a diffuse margin towards the duodenum and the peripancreatic fatty tissue. The common bile duct and the pancreatic duct both presented with high-grade stenosis. More than 100 enlarged lymph nodes were found in the specimens of small intestine and pancreas. The spleen (14.5 × 12 × 4.6 cm) appeared unremarkable.

**Fig. 3:**
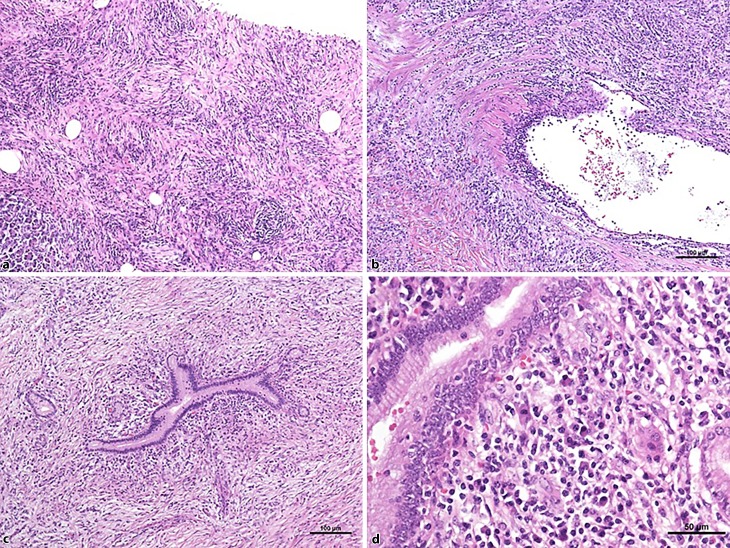
**a** Most of the pancreatic parenchyma (*lower left corner*) is replaced by fibrosis with a storiform pattern. **b** Numerous inflammatory cells infiltrate the wall of a venous vessel (venolitis). **c** Pancreatic duct surrounded by storiform fibrosis and inflammatory infiltrates. **d** The periductal infiltrate contains lymphocytes and numerous plasma cells

Histopathological findings included abundant areas with storiform fibrosis replacing parts of the pancreatic parenchyma with extension into the papilla, and partially surrounded the main and interlobular pancreatic ducts, which also showed irregular outlines (Fig. 3). The fibrous tissue was infiltrated mainly by lymphocytes and plasma cells, which were often centered around the pancreatic ducts. However, there was no granulocytic infiltration and IgG4 staining was negative. Further, histology of the pancreatic specimens revealed obliterative venulitis, neuritis and lymphadenopathy with interfollicular reactive hyperplasia and atrophic lymph follicles. Storiform fibrosis, stenosis and IgG4-negative periductal lymphoplasmacytic inflammation without granulocytic infiltration also affected the common bile duct. These histopathological findings supported the diagnosis of autoimmune pancreatitis (AIP) type 1, even without elevated numbers of IgG4-positive plasma cells, associated with fibrosis and atrophy of the exocrine tissue, and with stenosis of the pancreatic and common bile duct.

The AIP is a distinct form of pancreatitis with autoimmune features and frequently presents with painless obstructive jaundice (60–75%), a pancreatic mass or focal enlargement of the pancreas. Pancreatic insufficiency in the form of new or worsened hyperglycemia and steatorrhea may be present and, in a rare case, acute pancreatitis as well [21]. The disease is characterized by abnormalities of *h*istology, *i*maging, *s*erology, other *o*rgan involvement, and *r*esponse to (steroid) *t*herapy (HISORt criteria; [22]), or different combinations of these cardinal features [23]. The international consensus diagnostic criteria (ICDC) for AIP describe two subtypes: type 1 lymphoplasmacytic sclerosing pancreatitis (LPSP), and type 2 idiopathic duct-centric pancreatitis (IDCP) or AIP with granulocytic epithelial lesion (GEL) [24]. The characteristic histological features of both types of AIP are summarized in Table 2. Massive infiltration by IgG4 positive lymphocytes is a histological hallmark of AIP [25, 26] but is not pathognomonic for the disease and was not found in this case. Histological review is essential for diagnosis of AIP and differentiation from other forms of chronic pancreatitis such as chronic pancreatitis-not otherwise specified (CP-NOS), follicular pancreatitis, hereditary chronic pancreatitis, paraduodenal chronic pancreatitis and lobular fibrosis with low-grade Pan-IN.

**Table 2:** Criteria for autoimmune pancreatitis (AIP) types 1 and 2 [24]

Criterion	AIP type 1	
	*Level 1*	*Level 2*
Parenchymal imaging	Typical: diffuse enlargement with delayed enhancement (sometimes associated with rim-like enhancement)	Indeterminate (including atypical^a^): Segmental/focal enlargement with delayed enhancement
Ductal imaging (ERP)	Long (>1/3 length of the main pancreatic duct) or multiple strictures without marked upstream dilatation	Segmental/focal narrowing without marked upstream dilatation (duct size <5 mm)
Serology	IgG4 > 2 × upper limit of normal value	IgG4 1–2 × upper limit of normal value
Other organ involvement	a or b	a or b
	a. Histology of extrapancreatic organs, any 3 of the following:	a. Histology of extrapancreatic organs including endoscopic biopsies of bile duct^b^, both of the following:
	(I) Marked lymphoplasmacytic infiltration with fibrosis and without granulocytic infiltration	(I) Marked lymphoplasmacytic infiltration without granulocytic infiltration
	(II) Storiform fibrosis	(II) Abundant (>10 cells/HPF) IgG4-positive cells
	(III) Obliterative phlebitis	
	(IV) Abundant (>10 cells/HPF) IgG4-positive cells	
	b. Typical radiological evidence	b. Physical or radiological evidence
	At least 1 of the following:	At least 1 of the following:
	(I) Segmental/multiple proximal (hilar/intrahepatic) or proximal and distal bile duct stricture	(I) Symmetrically enlarged salivary/lachrymal glands
	(II) Retroperitoneal fibrosis	(II) Radiological evidence of renal involvement described in association with AIP
Histology of the pancreas	LPSP and at least 3 of the following:	LPSP and any 2 of the following:
	(I) Periductal lymphoplasmacytic infiltrate without granulocytic infiltration	(I) Periductal lymphoplasmacytic infiltrate without granulocytic infiltration
	(II) Obliterative phlebitis	(II) Obliterative phlebitis
	(III) Storiform fibrosis	(III) Storiform fibrosis
	(IV) Abundant (>10 cells/HPF) IgG4-positive cells	(IV) Abundant (>10 cells/HPF) IgG4-positive cells
Response to steroids^c^	Diagnostic steroid trial	
	Rapid (≤2 weeks) radiologically demonstrable resolution or marked improvement in pancreatic/extrapancreatic manifestations	
	AIP type 2	
	*Level 1*	*Level 2*
Parenchymal imaging	Typical: Diffuse enlargement with delayed enhancement (sometimes associated with rim-like enhancement)	Indeterminate (including atypical^a^): Segmental/focal enlargement with delayed enhancement
Ductal imaging (ERP)	Long (>1/3 length of the main pancreatic duct) or multiple strictures without marked upstream dilatation	Segmental/focal narrowing without marked upstream dilatation (duct size <5 mm)
Other organ involvement	–	Clinically diagnosed inflammatory bowel disease
Histology of the pancreas (core biopsy/resection)	IDCP:	
	Both of the following:	Both of the following:
	(I) Granulocytic infiltration of duct wall (GEL) with or without granulocytic acinar inflammation	(I) Granulocytic infiltration of duct wall (GEL) with or without granulocytic acinar inflammation
	(II) Absent or scant (0–10 cells/HPF) IgG4-positive cells	(II) Absent or scant (0–10 cells/HPF) IgG4-positive cells
Response to steroids^c^	Diagnostic steroid trial	
	Rapid (≤2 weeks) radiologically demonstrable resolution or marked improvement in manifestations	

*AIP* autoimmune pancreatitis, *ERP* endoscopic retrograde pancreatography, *LPSP* lymphoplasmacytic sclerosing pancreatitis, *HPF* high power field, *IDCP* idiopathic duct-centric pancreatitis

^a^atypical; some AIP cases may show low-density mass, pancreatic ductal dilatation or distal atrophy. Such atypical imaging findings in patients with obstructive jaundice and/or pancreatic mass are highly suggestive of pancreatic cancer. Such patients should be managed as pancreatic cancer unless there is strong collateral evidence for AIP and a thorough work-up for cancer is negative

^b^Endoscopic biopsy of the duodenal papilla is a useful adjunctive method because the ampulla is often pathological in AIP

^c^Diagnostic steroid trial should be conducted carefully by pancreatologists with caveats only after negative workup for cancer including endoscopic ultrasound-guided fine needle aspiration

### Dr. G.J. Krejs:

In the past, AIP has been described by various terms including chronic inflammatory sclerosis of the pancreas, chronic sclerosing pancreatitis, non-alcoholic duct-destructive chronic pancreatitis, lymphoplasmacytic sclerosing pancreatitis, idiopathic tumefactive chronic pancreatitis and idiopathic duct-centric chronic pancreatitis [27]. With an estimated prevalence of <1–2.2 per 100,000 in the general population, AIP is a rare disorder [28, 29]. Type 1 is the most prevalent subtype worldwide, accounting for up to 80% of cases in the USA and AIP type 2 is relatively more common in Europe but type 1 still remains the more prevalent subtype [30]. This disease is typically diagnosed later in life (80% of patients are over 50 years of age at diagnosis) with type 1 diagnosed an average of 16 years later than type 2 [31, 32]. In type 1 AIP males are more often affected than females (3:1; [21]), but there is no gender predilection documented for type 2 [33]. While type 2 AIP is a pancreas-specific disease mostly without extrapancreatic organ involvement and negative serology for IgG4 and autoantibodies, type 1 AIP is suggested to be a pancreatic manifestation of an IgG4-related disease characterized by systemic inflammation of unknown origin [27]. Despite the frequent association with elevated levels of IgG4 in serum and plasma cells in tissue, the role of IgG4 in AIP remains unclear. In fact, it is even unclear whether IgG4 type antibodies in AIP behave like tissue-destructive antibodies, i.e. autoantibodies, or are overexpressed in response to an unknown stimulus [23]. In healthy individuals IgG4 comprises 4–6% of the total IgG [34] and is considered a non-inflammatory antibody because of its relative inability to fix complement and its poor capacity to bind Fc receptors [35, 36]. Thus, IgG4 may be secondarily induced to dampen the extensive immune reaction in AIP [23]. In contrast, IgG4 has been demonstrated to be self-reactive in patients with IgG4-related disease [37], which supports the suggestion of primary IgG4 tissue-destructive properties in AIP [23]. A positive test for IgG4 is not pathognomonic for AIP or IgG4-related diseases and can occur in benign inflammatory processes such as allergic diseases, parasitic infestations and pemphigus vulgaris [34], as well as in malignancies such as pancreatic and bile duct cancers [23]. Although not disease-specific, IgG4 has the highest diagnostic value as a single serological test in AIP because elevated serum IgG4 is found in up to 75% of patients [38]; however, a subset of type 1 AIP patients who are seronegative for IgG4 should not be classified as type 2 [39]. Other serological findings in AIP include hypergammaglobulinemia, elevated IgG, antinuclear antibodies, anti-smooth muscle antibodies, carbonic anhydrase II antibodies, lactoferrin antibodies and rheumatoid factor [40, 41].

### Dr. P. Fickert:

The pathogenesis of AIP is not completely understood. Autoimmune processes are suggested to play a pivotal role in genetically susceptible patients. Human leukocyte antigen (HLA) DRB1*0405-DQB1*0401 haplotype, substitution of aspartic acid at DQB1 46 [42, 43] and single nucleotide polymorphism in cytotoxic T lymphocyte-associated antigen-4 (CTLA-4), which is expressed on CD4+ and CD8+ T cells and important for the regulation of T cell stimulation [44, 45], are discussed as key genetic factors in AIP. Moreover, *Helicobacter pylori* could probably trigger AIP via molecular mimicry in genetically predisposed individuals due to the significant homology between human carbonic anhydrase II and alpha-carbonic anhydrase of *Helicobacter pylori* with the homologous segments containing the binding motif of the HLA molecule DRB1*0405 [46]. Complement activation [47], Th2 cells and regulatory T cells (Treg) [48] are also hypothesized to be involved in the pathogenesis of AIP.

The clinical presentation of AIP is non-specific, sharing overlapping features with other diseases such as pancreatic cancer. Diagnosis requires a variable combination of histopathological, imaging and serological features in the presence of typical extrapancreatic lesions and a predictable response to steroids [27]. According to the ICDC, rapid response (≤2 weeks) to steroids is a diagnostic criterion for AIP (Table 2), but in some cases response may even take 1–4 months [49, 50]. Response rates have further been reported to vary between 50–75% and 99% in patients with type 1 and 92% in those with type 2 AIP [21, 49, 50]. Spontaneous resolution of AIP (in up to 30% of cases) [51] and intermittent courses as observed in this case have also been described without steroid therapy. The diagnosis can therefore be a real challenge for clinicians. Since type 1 AIP as in this case is considered to be part of an IgG4 systemic disease process, there are a number of associated extrapancreatic manifestations that occur in up to 77% of patients [52]. Extrapancreatic manifestations can be useful in the diagnosis of AIP and, therefore, are part of the HISORt criteria. The most common are hilar lymphadenopathy, sclerosing cholangitis, retroperitoneal fibrosis, salivary and lacrimal gland involvement and tubulointerstitial nephritis (summarized by [53]), worsening or onset of diabetes mellitus or symptoms such as back pain, weight loss and fatigue [40, 52]. Conditions less frequently reported are hypophysitis and chronic thyroiditis [54]. Abdominal pain is more common in patients with type 2 AIP (68%) than in those with type 1 (41%); inflammatory bowel disease is only observed in type 2 [24, 31, 40]. Biliary disease associated with jaundice is one of the most common extrapancreatic manifestations of AIP and mainly attributable to obstruction at the level of the intrapancreatic portion of the common bile duct, associated with an inflammatory pancreatic head mass. This condition occurring in 20–88% of cases of AIP has been termed IgG4-associated cholangitis (IAC) [55]. An overlap between IAC and PSC can be suggested because serum IgG4 levels are increased in 9–36% of patients with PSC but only in 1% of patients with other liver diseases [56, 57]. Interestingly, PSC patients with elevated serum IgG4 levels have a more rapid disease progression than those with normal levels [55].

This patient showed several extrapancreatic manifestations including lymphadenopathy, sclerosing cholangitis, jaundice and fatigue. Moreover, he complained of pruritus and skin manifestations that had been seen by the consulting dermatologist, Dr. Wutte.

### Dr. N. Wutte:

**Fig. 4:**
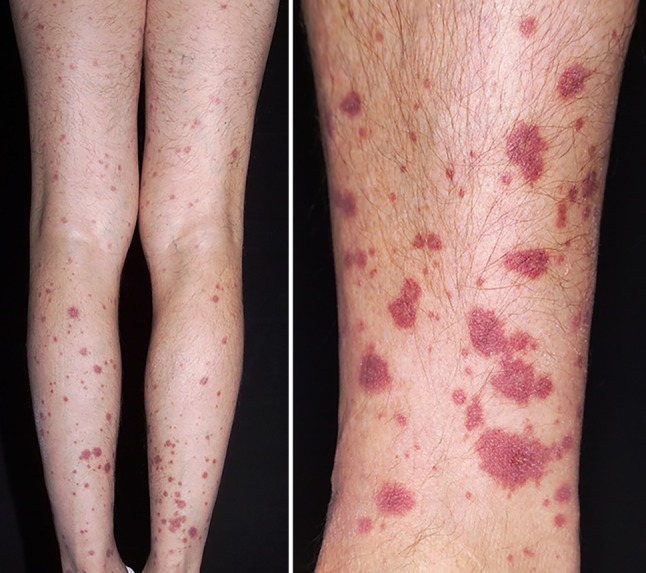
Purpuric, partly confluent plaques up to 1.5 cm in size on the lower extremities with central bullous transformation in larger lesions

**Fig. 5:**
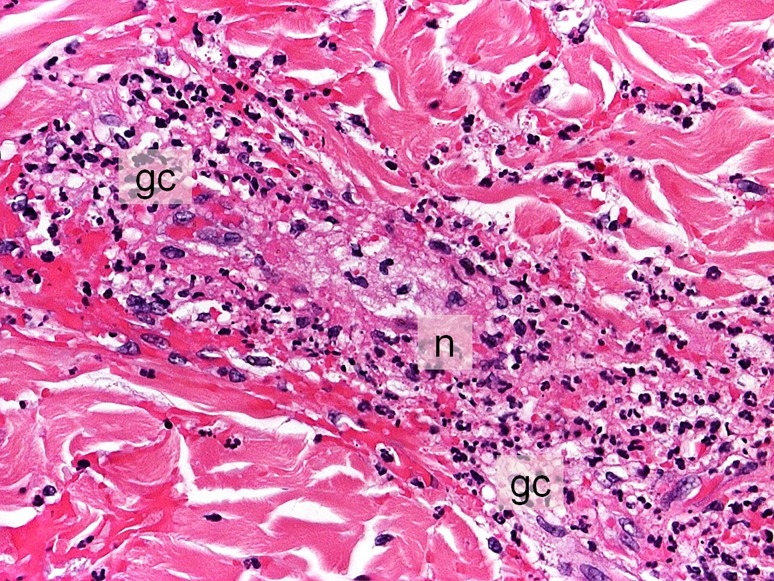
Skin biopsy showing leukocytoclastic vasculitis. Perivascular infiltrate of neutrophilic and eosinophilic granulocytes (*gc*) with focal leukocytoclasis in the superficial dermis, fibroid necrosis of the vessel walls (*n*)

The patient was first seen at the outpatient dermatological clinic of the University Medical Center in Graz a few days prior to total pancreatectomy because he had rapidly developed exanthemous lesions on the lower extremities. The purpuric plaques were sharply delineated at the sock line, had a diameter of up to 3 cm and were reaching up the legs, but without vesication or necrosis (Fig. 4). The patient was diagnosed with leukocytoclastic vasculitis and successfully treated with local corticosteroids. As there were repeated relapses during the following year, a biopsy was performed to confirm the diagnosis of leukocytoclastic vasculitis (Fig. 5). Leukocytoclastic vasculitis is an immune complex mediated disorder with degradation of leukocytes and subsequent destruction of small postcapillary venules. Clinically, leukocytoclastic vasculitis is characterized by palpable purpuric lesions. In children the most common type of vasculitis is IgA-mediated, in adults IgM and IgG-mediated vasculitis is more prevalent. The disease can be caused by specific medication, infections, tumors or hematologic diseases and is frequently observed in autoimmune diseases; however, in up to 50% of patients the underlying cause of immune-mediated leukocytoclastic vasculitis remains unclear [58, 59]. Cutaneous small vessel vasculitis in association with AIP is rare and was first documented by Garg et al. in a patient with IgG4 positive AIP type 1 [60]. General evidence in support of the association between extrapancreatic manifestations and AIP include (1) shared characteristic histopathological findings of lymphoplasmacytic infiltration, IgG4-positive plasma cell infiltration, obliterative phlebitis and storiform fibrosis, (2) frequent co-existence or occurrence, (III) predictably favorable response to steroids and (4) differentiation from the lesion of the corresponding organ [27]. The immunopathological mechanism between AIP and cutaneous purpuric leukocytoclastic vasculitis is not clear but an increased deposition of immune complexes formed in response to the underlying systemic disease is a possible explanation; however, leukocytoclastic vasculitis may even occur without demonstrable histological immunoreactivity [61]. The discussed IgG4 negative patient reported repeated relapses of the purpuric rash with intermittent improvement. This might have been due to physical activity consistently identified as a precipitating and/or aggravating factor in leukocytoclastic vasculitis [61].

### Dr. P. Fickert:

At a follow-up visit one year after total pancreatectomy, the patient’s liver function tests were all normal.

### Dr. M. Peck-Radosavljevic:

This case shows that making the correct diagnosis and differentiating AIP from other diseases, especially pancreatic cancer, is of utmost importance. Use of standard diagnostic criteria will usually allow AIP to be distinguished from pancreatic cancer. In some patients, pancreatic biopsy, a therapeutic trial with steroids, or surgery (as in the discussed case) will be needed to clarify the diagnosis of AIP.

## Final diagnosis

Autoimmune pancreatitis type 1 (IgG4 negative) with associated leukocytoclastic vasculitis.
